# Cancer Control in Low- and Middle-Income Countries: Is It Time to Consider Screening?

**DOI:** 10.1200/JGO.18.00200

**Published:** 2019-03-25

**Authors:** Shailja C. Shah, Violet Kayamba, Richard M. Peek, Douglas Heimburger

**Affiliations:** ^1^ Vanderbilt University Medical Center, Nashville, TN; ^2^ University of Zambia School of Medicine, Lusaka, Zambia

## Abstract

The rising prevalence of noncommunicable diseases globally, with a strikingly disproportionate increase in prevalence and related mortality in low- and middle-income countries (LMICs), is a major threat to sustainable development. The epidemiologic trend of cancers in LMICs is of particular concern. Despite a lower incidence of cancer in LMICs compared with high-income countries, total cancer-related mortality is significantly higher in LMICs, especially in people younger than 65 years of age. The enormous economic impact of premature mortality and lost productive life years highlights the critical importance of galvanizing cancer prevention and management to achieve sustainable development. The rising burden of cancer in LMICs stresses an already weak health care and economic infrastructure and poses unique challenges. Although the WHO acknowledges that the effective management of cancer relies on early detection, accurate diagnosis, and access to appropriate multimodal therapy, the placement of priority on early detection cannot be assumed to be effective in LMICs, where limited downstream resources may be overwhelmed by the inevitable increases in number of diagnoses. This review discusses several factors and considerations that may compromise the success of cancer control programs in LMICs, particularly if the focus is only on early detection through screening and surveillance. It is intended to guide optimal implementation of cancer control programs by accentuating challenges common in LMICs and by emphasizing the importance of cancer prevention where relevant so that communities and stakeholders can work together to devise optimal means of combatting the growing burden of cancer.

## INTRODUCTION

The end of 2015 marked the culmination of the Millennium Development Goals and the inauguration of the even more ambitious Sustainable Development Goals (SDGs). An overarching theme of these goals is to fight inequality across all realms, including social, environmental, and economic.^1^ Noncommunicable diseases (NCDs) were identified as one major challenge to sustainable development. Implementation of the Millennium Development Goals decreased the burden of group 1 causes of mortality (pregnancy- and childbirth-related issues, infant mortality, nutritional deficiencies, and communicable diseases,^2^ albeit still the main drivers of mortality in low- and middle-income countries [LMICs]),^3^ but this has been countered by a steadily increasing prevalence of NCDs and related mortality. The majority of deaths globally are now due to NCDs, with cancer responsible for at least 20% of all mortality.^4^ Although the overall incidence of cancer is lower in LMICs compared with high-income countries (HICs), total cancer-related mortality is significantly higher in LMICs, especially for people younger than 65 years of age; the greater economic impact as a result of premature mortality and lost years of productivity is especially problematic for these countries.^5-7^ In 2015, 78% of all global deaths attributable to NCDs, including cancer, occurred in LMICs, with nearly 50% of deaths in LMICs considered to be premature.^2^

The rising cancer burden in LMICs stresses already weak health care and economic infrastructures and poses unique challenges, particularly because extrapolation of the experiences of cancer control programs in HICs to LMICs is often inappropriate. The rationale for cancer control programs that prioritize screening and surveillance to increase the likelihood of early cancer diagnosis^8^ not only assumes that the undiagnosed cancer would have been the underlying cause of death but also assumes adequate availability of downstream resources to appropriately attend to and manage the increased number of preclinical cases diagnosed. Accordingly, although a decrease in cancer incidence and cancer-related mortality is the intended outcome of cancer control programs in LMICs, this might not be the actual experience.

**CONTEXT**

**Key Objective**
Although the core concepts of cancer prevention and control programs overlap between low- and middle-income countries (LMICs) and high-income countries, effective implementation and execution of such programs in LMICs necessitates distinct considerations, which are discussed in this review.
**Knowledge Generated**
Prioritizing early detection through screening/surveillance, as done in high-income countries, cannot be assumed to be effective in LMICs, where limited downstream resources for treatment may be overwhelmed by the expectedly increased number of cancer diagnoses. Careful attention must be paid to ensure that all aspects of cancer control programs are balanced to limit unintended harm.
**Relevance**
The growing burden of cancer in LMICs with disproportionately poor outcomes requires urgent attention. The implementation and continued development of cancer prevention and control programs in LMICs must be an iterative process with realistic expectations and interventions tailored to the specific population, its cultural values and beliefs, and its health care and economic infrastructure.

Although the WHO acknowledges that effective cancer treatment relies on early detection, accurate diagnosis, and access to multimodal cancer therapy, emphasis is placed on early detection as a lynchpin to cancer control in LMICs.^8^ In LMICs, where resources are already constrained and access to health care is far from universal, careful attention is needed to ensure that the intended outcomes of cancer control programs are achieved and that the balance of benefits to potential harms is favorable. This review highlights factors that may compromise the success of cancer control programs in LMICs that emphasize early cancer detection as opposed to, for example, a focus on cancer prevention or risk reduction. It is intended neither to temper nor dissuade enthusiasm for meeting the challenges of the growing cancer burden in LMICs but, rather, to serve as a guide to help to focus and optimize cancer control programs and bring to the surface challenges that are unique to LMICs so that they can be anticipated and addressed.

## THE BURDEN OF CANCER IN LMICS

In 2018, there was an estimated 18.1 million new cancer diagnoses and 9.6 million cancer deaths.^9,10^ The WHO estimates that by 2040, this will increase to 29.5 million new cancer diagnoses and 16.5 million cancer-related deaths annually.^11^ As noted, although HICs have overall higher cancer incidence rates, mortality rates and total mortality as a result of cancer are significantly higher in LMICs and continue to rise, whereas mortality rates in HICs are either decreasing or stable.^5^ In 2012, 65% of all cancer deaths globally occurred in LMICs,^10,12^ an estimate that is projected to increase to 75% by 2030.^11,13^ Reasons for these disparate trends include better risk factor control in HICs (lower infection-associated cancers, antismoking campaigns, other preventive measures),^14-16^ educational resources, increased number of screening and surveillance programs with earlier detection of disease, and improved cancer therapies. LMICs have been experiencing increasing cancer-related mortality as a result of rising obesity rates; increasingly sedentary lifestyles; dietary factors; excess tobacco and alcohol use; and persistent carcinogenic infections like *Helicobacter pylori*, hepatitis B virus, and human papilloma virus, not to mention other contributing factors that are less well understood.^7,17^ Not surprisingly, LMICs share a disproportionate burden of infection-associated cancer mortality, including gastric cancer, hepatocellular carcinoma, and cervical cancer, in striking contrast to HICs, where infection-related cancer mortality is rare. Effective vaccination and *H pylori* eradication therapy represent critical opportunities for a significant reduction of the global cancer burden.^14,18,19^ Of the 14 million new cancer diagnoses globally in 2012, 15.4% overall were attributable to infectious agents.^7,20^ Indeed, the population-attributable percentage was significantly higher in less-developed countries than in developed countries, with some countries in sub-Saharan Africa having greater than 50% attributable fractions related to infectious agents versus less than 5% in the United States and Canada.^7^ Especially striking is that up to one third of infection-attributable cancers arise in people younger than age 50 years, which partially accounts for the excessive premature deaths as a result of cancer in LMICs and further highlights the economic burden. The burden of cancer in LMICs may even be an underestimate because it is rare for LMICs to have reliable cancer registries and reporting systems.^21^

## ADDRESSING THE BURDEN

According to projected population statistics, these global patterns and trends are expected to worsen in the future. With this motivation and in the spirit of the SDGs, the WHO introduced the comprehensive Global Action Plan for the Prevention and Control of NCDs 2013-2020.^22^ Unfortunately, although no cancer-specific metrics are included in the plan, the WHO recognizes four key components to cancer control: prevention, early detection and diagnosis, treatment, and palliation. Inadequacies in these areas in LMICs undermine the efficacy and sustainability of cancer control programs in already resource-limited environments.

## KEY PRINCIPLES FOR CANCER SCREENING

A screening program must be acceptable, equitable, accessible, sustainable, and economically efficient for the target population, and the health care infrastructure must be equipped to manage the increased case finding with respect to treatment, support, and follow-up. Without reasonable assurance that each of these is met, implementation of a screening program may be premature, and resources allocated for cancer control should instead focus on improvements in risk factor reduction and improved health literacy as well as on cancer treatment and palliation where appropriate. With gradual improvements in health care infrastructure in parallel, cancer control programs, including those that emphasize screening and surveillance, have had the highest likelihood of achieving the intended goal without imposing unnecessary risk, psychological stress, compromised quality of life, and financial hardship for patients and their families.

The purpose of cancer screening is to detect either precancerous lesions or preclinical cancer at a stage when therapeutic interventions are associated with better disease outcomes, such as cancer prevention and reduced cancer-related mortality, respectively. At the core, the benefits of screening for a particular cancer must outweigh the associated risks of screening. The success of screening interventions also depends on the disease burden; availability of a test with appropriately robust statistical performance; acceptability to the population of interest; ease of use/reliability of the test; population participation in the program; and critically, adequate resources and human power to appropriately manage diagnosed patients and provide appropriate, ongoing follow-up care. The WHO has established 10 key principles when considering any screening test^23^ (Table 1). Consideration of these criteria highlights potential challenges and barriers that might compromise the success of screening programs in LMICs compared with resource-replete countries.

**TABLE 1:**
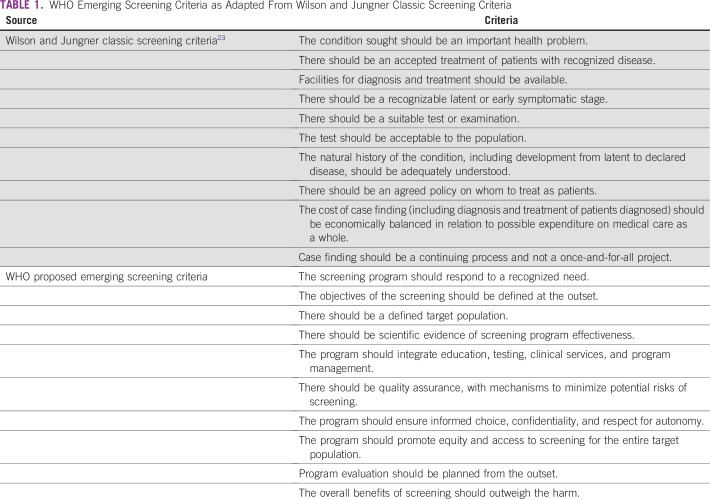
WHO Emerging Screening Criteria as Adapted From Wilson and Jungner Classic Screening Criteria

Furthermore, harms of screening should incorporate not only traditionally assumed harms in the physical sense but also the psychological and financial consequences of screening. For example, the psychological impact of a false-positive screening test and the financial impact of extraneous downstream diagnostic and therapeutic interventions are relevant. Potential stigma from a cancer diagnosis and consequences of cancer treatment, such as hair loss and infertility or mastectomy for breast cancer, have been incompletely evaluated (or not evaluated at all) in LMICs for their psychological impact; with consideration of the gamut of cultural beliefs and societal norms encompassed across LMICs, these are likely to differ among countries and communities.^24-26^

An effective screening program relies on participation in screening by the majority of the target population^23^ and highlights the importance of a diagnostic test that is accessible and acceptable to the population of interest. For example, an upper endoscopy for esophageal or gastric cancer screening not only may be inaccessible to the population (or accessible only to a small proportion) but also may meet with important societal and cultural barriers that limit widespread participation. As another example, the acceptability of cervical cancer screening has limited its uptake in some cultures.^27^ General public awareness of cancer screening and its potential benefits also may be lacking, hand-in-hand with an often-ingrained belief that all cancer is incurable and universally fatal.^28,29^ Educational initiatives and community empowerment are important adjuncts to the successful implementation of screening and surveillance programs.

## STRUCTURE OF CANCER CARE IN LMICS

Data from LMICs unfortunately are limited when it comes to the current status of cancer care and infrastructure, particularly because health care infrastructures in these settings were historically built around addressing communicable diseases, nutritional deficiencies, and maternal-child health. With cancer and other NCDs, significant resource utilization is also expected after the immediate diagnostic and treatment phase because cancer tends to recur, especially if initial treatment is suboptimal. The rise of NCDs poses unique challenges and requires horizontal integration of the systems currently in place, with new systems and services focused on cancer control in LMICs. This requires collaboration on the international, national, and locoregional level^30^; fortunately, there has been a renewed commitment by global health agencies to address the unmet need for cancer prevention and control in LMICs.^31^

Four key priorities have been identified to promote health services for cancer control and data acquisition: capacity building in oncologic health services research, policy, and planning relevant to LMICs^31^; development of high-quality health data sources, such as population-based cancer registries, to identify the process and outcomes of cancer management to ensure that they are iterative and achieve quality cancer control^30,32^; more oncology-related economic evaluations in LMICs^33^; and exploration of high-quality models of cancer control in LMICs as opposed to the extrapolation of experiences from HICs. Unfortunately, making headway in these four interrelated areas requires money, improved policy, and increased transparency. An estimated 0.1% of total health care expenditure should be dedicated to health services and policy research in LMICs, but on average, the amount currently spent is approximately 0.007% of total health care expenditures in LMICs.^33^ Also not surprising is that the majority of LMICs do not have adequate cancer registries. In the International Agency for Research on Cancer report on global cancer incidence, only 1% of Africa, 4% of Asia, and 4% of South and Central America have population-based data sufficient for inclusion compared with 80% of North America.^32^ Although implementation of a comprehensive health information system is estimated to be a cost-effective intervention in LMICs^34^ and is critical to ensuring the success and efficacy of integrated, comprehensive national cancer prevention and control plans, the upfront costs represent a significant expenditure and barrier.

### Cancer Treatment in LMICs: Medical Therapy

Alongside overall infrastructure considerations, attention must be paid to cancer therapy; specifically as it relates to availability; accessibility; efficacy; safety; and of similar importance, post-therapy monitoring and follow-up. Unfortunately, data that inform therapeutic decision making for cancer management in HICs might not always be applicable in LMICs. Aside from perhaps radiotherapy, reliable data on the outcomes of cancer therapy in LMICs are essentially nonexistent. It is standard of care for chemotherapeutic agents to be tested and their outcome data scrutinized with respect to efficacy and safety before being offered to patients in HICs; even then, administration of anticancer therapy requires the careful care and oversight of a multidisciplinary team to manage any adverse effects or complications of therapy as well as to monitor the tumor’s response to therapy (eg, radiographically as with computed tomography, magnetic resonance imaging, and positron emission tomography scanning or a combination). Such multimodal care is scarce in LMICs, and thus, the risk-benefit ratio of chemotherapy offered in HICs is distinct from that in real practice in LMICs. Some LMICs do not have the capacity to perform rigorous clinical trials to assess their own therapeutic outcomes, so little information exists to guide therapeutic management of diagnosed cancers. Selection of appropriate therapy often requires key prerequisite investigations, such as the identification of hormone receptor status; otherwise, the therapy may be ineffective and wasteful or worse, lead to adverse psychological and financial consequences for the patient and family members. In addition, whether these chemotherapeutic agents have adequate performance in populations where the tumor biology, patient-related characteristics, or specific environmental determinants may differ from the HIC populations in which they were initially tested and approved has not been investigated.

Little is known about the capacity of specific LMICs to meet the complex network of challenges that accompany rising cancer incidence in an aging population with respect to both human capacity, such as medical and surgical specialists and appropriately trained nurses and pharmacists (ie, to prepare and administer anticancer therapies safely),^35^ and resource capacity, such as adequate hospital beds (including isolation wards for immunocompromised patients with cancer), antibiotics (including extended-spectrum antibiotics and those for opportunistic infections), adequate imaging modalities for diagnosis and follow-up monitoring, supportive therapies (including blood transfusions and bone marrow stimulants for bone marrow toxicity after radiation or chemotherapy), among other capacities in the routine care of patients undergoing cancer treatment in non–resource-limited settings. Complications such as neutropenic fever, infections, and blood clots, among others, are feared consequences, but are not unexpected with anticancer therapy. These consequences that are routinely managed on oncology hospital wards in HICs must be taken seriously because the risk of anticancer therapy may rapidly outweigh the benefit in LMICs if, for example, serious infections and sepsis routinely occur that cannot be managed appropriately or are cost prohibitive.

Although the exact availability and types of anticancer therapies in LMICs are unknown, a WHO survey found that only 22% of African countries and 43% of Southeast Asian countries report availability of anticancer therapy, with the specific therapies not specified; this is in marked contrast to a reported availability that exceeds 90% in Europe.^36^ With recognition of the challenge of affordable anticancer therapy, the WHO has strived to increase access to cytotoxic therapies on its Model Lists of Essential Medicines beyond those select therapies primarily appropriate for childhood cancers like Burkitt’s lymphoma.^37^ Drug shortages are common and encompass both patented and generic drugs.^37^ Indeed, even when therapy is available and effective, cost remains an overriding concern. Even the most common medications, such as antibiotics and antinausea medications, may be inaccessible for families because of cost. A report by the WHO found that 20% to 60% of health expenditures in developing and transitional countries are for medicines, which is significantly more than in developed countries.^38,39^ After food, medicines are the single largest out-of-pocket expenditure of families in developing countries.^39^ Achievement of the provisions of the third SDG (achieve universal health coverage) hopefully will address this, at least in part, although where cancer treatment falls within this stipulation is uncertain.

### Cancer Treatment in LMICs: Surgical Therapy and Radiotherapy

Cancer treatment requires a multimodal and tailored approach because not all cancers are biologically similar. Some require a three-pronged approach with surgical resection, chemotherapy, and radiotherapy for durable remission and, ideally, cure, whereas others may be cured with surgery alone. Unfortunately, access to surgical services, let alone affordable surgical services, is not an option for the majority of the world’s population, even though it may make the difference between curing a cancer and succumbing to an otherwise curable disease. An estimated 5 billion people—the majority of the world’s population—lack access to safe, affordable surgical services when needed, not to mention appropriate accompanying anesthesia care.^40^ These numbers may be much higher when considering those who would benefit from surgical resection of cancer, although not always strictly considered life-saving. In addition, an estimated 33 million people globally face financial ruin from payments for surgery and anesthesia per year. The Lancet Commission on Global Surgery published a landmark initial report entitled Global Surgery 2030 that highlights the current deficiencies and implores policymakers, implementers, and funders to include core indicators and associated targets for universal access to safe and affordable surgical and anesthesia care by 2030.^40^ If this is achieved, then outcomes of screening for and early detection of cancers for which surgical resection is appropriate may be shifted such that the benefit of screening outweighs the risks. Also of utmost importance is addressing cultural barriers and societal norms that may limit the acceptability of and participation in surgical procedures. If not addressed, these may impede successful implementation of key aspects of cancer control programs.

The accessibility of radiotherapy is also inadequate to meet the needs of those who would benefit from services. One study estimated that the supply of radiotherapy machines in Africa was sufficient to meet only 18% of the radiation needs, and 22 African and Asian countries did not have access to radiotherapy at all.^41^ Whereas developed countries have one radiotherapy machine per 250,000 people, developing countries have one per 7 million.^42^ Furthermore, 5 million new people annually are estimated to need radiation therapy in LMICs.^42^

### Cancer Treatment in LMICs: Palliative Care and Pain Control

Although palliative care is an underused resource in HICs, particularly in the United States, it is an all-too-often unavailable resource in LMICs. The majority of cancers in LMICs are diagnosed in the advanced stage with limited therapeutic options, even in the event that they are available and affordable. As such, the WHO recognizes palliation as the fourth key principle of adequate cancer control in LMICs. Palliative care improves the quality of life of both patients and their families and takes a holistic approach by attending to physical, psychosocial, and cultural aspects. Unfortunately, of the 40 million people in need of palliative care, nearly 80% reside in LMICs, a number likely to increase in the coming years.^43^ Although beyond the scope of this review, a few key factors that underlie the insufficient palliative care services available in LMICs are worth mentioning because the consequences of this unmet need have a profound, negative impact on the quality of life of many low-income populations. Morphine and other opiates critical to adequate relief from malignant pain are highly regulated and even unavailable in some countries as a result of government bans.^43^ According to a Human Rights Watch report in 2008, India’s morphine supply was adequate to cover only 4% of people who needed it.^44^ Hand-in-hand with these regulations are societal and cultural beliefs around pain and opiate use and the prominent shortage of professionals trained in palliative care, not to mention an overall lack of awareness and appreciation of the role of palliative care in terminal illness.^35,45^ An additional challenge is the level of supportive services needed by patients and their families, which depends on several variables, including the cancer behavior, response to therapy, and the family’s support system and financial capacity, among others.

## NEXT STEPS

Investment in cancer prevention and control on the broader scale is needed now more than ever in the face of an aging population in LMICs and rising cancer incidence and mortality. Attention should be paid to all four areas identified by the WHO as integral to the success of cancer control programs: risk factor modification and prevention, early diagnosis, treatment, and palliation. If existing treatments are not effective or are inaccessible, be it surgical, cytotoxic medical therapy, and/or radiotherapy, or the infrastructure too inadequate to manage expected and unexpected toxicities of therapies, the stage at which the cancer is diagnosed has little bearing on mortality. Moreover, early diagnosis of cancer, if appropriate management is not a realistic option, may negatively affect patient and family quality of life and financial stability and may even hasten death.

Some cancers in LMICs are preventable (or their risk significantly attenuated) either by eradication of or vaccination against carcinogenic infectious agents or by avoiding carcinogenic exposures, such as tobacco smoke or air pollutants from indoor cooking.^9,14,16,19,20,46^ Although this is not the case for the majority of cancers, campaigns focused on prevention are potentially relatively low-cost, high-impact interventions that are a positive step toward reducing global cancer burden. Historically, risk factor modification efforts have not enjoyed the same successes in LMICs as they have in HICs, and the reasons for this are several, including cultural barriers and infrastructure. Research focused on understanding and addressing these barriers may be informative and effective.

Any cancer control effort would benefit from rigorous testing in LMICs because evidence for interventions shown to be beneficial in resource-replete settings are not universally extrapolatable to resource-constrained settings.^47^ Evaluations should be iterative with continual re-evaluation and identification of successes and failures to inform the subsequent efforts, particularly because the majority of health care infrastructures in LMICs were developed in response to controlling communicable diseases, malnutrition, and maternal-child health.

In conclusion, cancer is a complex and growing health problem in LMICs that requires integration of multiple sectors that not only include but also extend beyond health care delivery. Cancer control efforts must always ensure that harms, including physical, psychological, and financial, are minimized and that the benefits outweigh the aggregate risks.

## References

[1] United Nations About the Sustainable Development Goals http://www.un.org/sustainabledevelopment/sustainable-development-goals

[2] WHO The top 10 causes of death http://www.who.int/mediacentre/factsheets/fs310/en/index1.html

[3] The World Bank World Bank Country and Lending Groups https://datahelpdesk.worldbank.org/knowledgebase/articles/906519

[4] WHO NCD mortality and morbidity https://www.who.int/gho/ncd/mortality_morbidity/en

[5] Torre LA Siegel RL Ward EM et al Global cancer incidence and mortality rates and trends--an update Cancer Epidemiol Biomarkers Prev 25 16 27 2016 26667886 10.1158/1055-9965.EPI-15-0578

[6] Sankaranarayanan R Screening for cancer in low- and middle-income countries Ann Glob Health 80 412 417 2014 25512156 10.1016/j.aogh.2014.09.014

[7] Plummer M de Martel C Vignat J et al Global burden of cancers attributable to infections in 2012: A synthetic analysis Lancet Glob Health 4 e609 e616 2016 27470177 10.1016/S2214-109X(16)30143-7

[8] WHO Guide to cancer early diagnosis https://www.who.int/cancer/publications/cancer_early_diagnosis/en

[9] Bray F Ferlay J Soerjomataram I et al Global cancer statistics 2018: GLOBOCAN estimates of incidence and mortality worldwide for 36 cancers in 185 countries CA Cancer J Clin 68 394 424 2018 30207593 10.3322/caac.21492

[10] International Agency for Research on Cancer Global Cancer Observatory https://gco.iarc.fr

[11] International Agency for Research on Cancer Cancer Tomorrow https://gco.iarc.fr/tomorrow/home

[12] Parkin DM Bray F Ferlay J et al Global cancer statistics, 2002 CA Cancer J Clin 55 74 108 2005 15761078 10.3322/canjclin.55.2.74

[13] The Lancet GLOBOCAN 2018: Counting the toll of cancer Lancet 392 985 2018 30264708 10.1016/S0140-6736(18)32252-9

[14] Danaei G Vander Hoorn S Lopez AD et al Causes of cancer in the world: Comparative risk assessment of nine behavioural and environmental risk factors Lancet 366 1784 1793 2005 16298215 10.1016/S0140-6736(05)67725-2

[15] Forouzanfar MH Alexander LT Anderson HR et al Global, regional, and national comparative risk assessment of 79 behavioural, environmental and occupational, and metabolic risks or clusters of risks in 188 countries, 1990-2013: A systematic analysis for the Global Burden of Disease Study 2013 Lancet 386 2287 2323 2015 26364544 10.1016/S0140-6736(15)00128-2 PMC4685753

[16] WHO WHO report on the global tobacco epidemic 2008 http://www.who.int/tobacco/mpower/2008/en

[17] de Martel C Ferlay J Franceschi S et al Global burden of cancers attributable to infections in 2008: A review and synthetic analysis Lancet Oncol 13 607 615 2012 22575588 10.1016/S1470-2045(12)70137-7

[18] Plummer M Franceschi S Vignat J et al Global burden of gastric cancer attributable to *Helicobacter pylori* Int J Cancer 136 487 490 2015 24889903 10.1002/ijc.28999

[19] Maucort-Boulch D de Martel C Franceschi S et al Fraction and incidence of liver cancer attributable to hepatitis B and C viruses worldwide Int J Cancer 142 2471 2477 2018 29388206 10.1002/ijc.31280

[20] International Agency for Research on Cancer Cancers attributable to infections https://gco.iarc.fr/causes/infections/home

[21] Siddiqui AH Zafar SN Global availability of cancer registry data J Glob Oncol 10.1200/JGO.18.00116 PMC6223496 30085880

[22] WHO Global action plan for the prevention and control of NCDs 2013-2020 http://www.who.int/nmh/publications/ncd-action-plan/en

[23] Andermann A Blancquaert I Beauchamp S et al Revisiting Wilson and Jungner in the genomic age: A review of screening criteria over the past 40 years Bull World Health Organ 86 317 319 2008 18438522 10.2471/BLT.07.050112 PMC2647421

[24] Lantz PM Dupuis L Reding D et al Peer discussions of cancer among Hispanic migrant farm workers Public Health Rep 109 512 520 1994 8041851 PMC1403528

[25] Ajekigbe AT Fear of mastectomy: The most common factor responsible for late presentation of carcinoma of the breast in Nigeria Clin Oncol (R Coll Radiol) 3 78 80 1991 2031886 10.1016/s0936-6555(05)81167-7

[26] Ekortarl A Ndom P Sacks A A study of patients who appear with far advanced cancer at Yaounde General Hospital, Cameroon, Africa Psychooncology 16 255 257 2007 17310465 10.1002/pon.1144

[27] Moreira ED Jr Oliveira BG Ferraz FM et al Knowledge and attitudes about human papillomavirus, Pap smears, and cervical cancer among young women in Brazil: Implications for health education and prevention Int J Gynecol Cancer 16 599 603 2006 10.1111/j.1525-1438.2006.00377.x 16681732

[28] Soliman AS Raouf AA Chamberlain RM Knowledge of, attitudes toward, and barriers to cancer control and screening among primary care physicians in Egypt: The need for postgraduate medical education J Cancer Educ 12 100 107 1997 9229273 10.1080/08858199709528463

[29] Imam SZ Rehman F Zeeshan MM et al Perceptions and practices of a Pakistani population regarding cervical cancer screening Asian Pac J Cancer Prev 9 42 44 2008 18439071

[30] Hanna TP Kangolle ACT Cancer control in developing countries: Using health data and health services research to measure and improve access, quality and efficiency BMC Int Health Hum Rights 10 24 2010 20942937 10.1186/1472-698X-10-24 PMC2978125

[31] Chan M Kazatchkine M Lob-Levyt J et al Meeting the demand for results and accountability: A call for action on health data from eight global health agencies PLoS Med 7 e1000223 2010 20126260 10.1371/journal.pmed.1000223 PMC2811154

[32] Cancer incidence in five continents. Volume IX IARC Sci Publ 160 1 837 2008 19388204

[33] Gonzalez Block MA Mills A Assessing capacity for health policy and systems research in low and middle income countries Health Res Policy Syst 1 1 2003 12646072 10.1186/1478-4505-1-1 PMC151554

[34] Stansfield SK Walsh J Prata N et al Information to improve decision making for health Jamison DT Breman JG Measham AR et al Disease control priorities in developing countries Washington, DC World Bank 2006

[35] Oulton JA The global nursing shortage: An overview of issues and actions Policy Polit Nurs Pract 7 34S 39S 2006 17071693 10.1177/1527154406293968

[36] Alwan A Maclean D Mandil A Assessment of national capacity for noncommunicable disease prevention and control: The report of a global survey, 2001 https://apps.who.int/iris/bitstream/handle/10665/67305/WHO_MNC_01.2.pdf?sequence=1&isAllowed=y

[37] Robertson J Barr R Shulman LN et al Essential medicines for cancer: WHO recommendations and national priorities Bull World Health Organ 94 735 742 2016 27843163 10.2471/BLT.15.163998 PMC5043203

[38] Organisation for Economic Co-operation and Development Drug spending in OECD countries up by nearly a third since 1998, according to new OECD data http://www.oecd.org/health/drugspendinginoecdcountriesupbynearlyathirdsince1998accordingtonewoecddata.htm

[39] WHO The World Medicines Situation Report http://www.who.int/medicines/areas/policy/world_medicines_situation/en

[40] Meara JG Leather AJM Hagander L et al Global surgery 2030: Evidence and solutions for achieving health, welfare, and economic development Lancet 386 569 624 2015 25924834 10.1016/S0140-6736(15)60160-X

[41] Barton MB Frommer M Shafiq J Role of radiotherapy in cancer control in low-income and middle-income countries Lancet Oncol 7 584 595 2006 16814210 10.1016/S1470-2045(06)70759-8

[42] International Atomic Energy Agency A silent crisis: Cancer treatment in developing countries, 2003 https://inis.iaea.org/search/search.aspx?orig_q=RN:35024620

[43] WHO Palliative care http://www.who.int/mediacentre/factsheets/fs402/en

[44] Human Rights Watch Unbearable pain: India’s obligation to ensure palliative care https://www.hrw.org/report/2009/10/28/unbearable-pain/indias-obligation-ensure-palliative-care

[45] Lamas D Rosenbaum L Painful inequities—palliative care in developing countries N Engl J Med 366 199 201 2012 22256803 10.1056/NEJMp1113622

[46] McCarthy WJ Meza R Jeon J et al Chapter 6: Lung cancer in never smokers: Epidemiology and risk prediction models Risk Anal 32 S69 S84 2012 22882894 10.1111/j.1539-6924.2012.01768.x PMC3485693

[47] Andrews B Semler MW Muchemwa L et al Effect of an early resuscitation protocol on in-hospital mortality among adults with sepsis and hypotension: A randomized clinical trial JAMA 318 1233 1240 2017 28973227 10.1001/jama.2017.10913 PMC5710318

